# *Nutrients*: 15th Anniversary

**DOI:** 10.3390/nu18091401

**Published:** 2026-04-29

**Authors:** Annalisa Noce, Jay R. Hoffman, Ines Gonzalez-Casanova

**Affiliations:** 1 UOSD Nephrology and Dialysis, Policlinico Tor Vergata, 00133 Rome, Italy; 2 Department of Systems Medicine, University of Rome Tor Vergata, 00133 Rome, Italy; 3 School of Health Sciences, Ariel University, Ariel 40700, Israel; jayho@ariel.ac.il; 4 Department of Applied Health Science, School of Public Heath, Indiana University, Bloomington, IN 47405, USA; inegonza@iu.edu

Proper nutrition is a fundamental determinant of human health and is structured through the intake of various nutritional components: (i) macronutrients, including carbohydrates, lipids, and proteins, which provide energy and essential structural materials for metabolic and physiological processes; (ii) micronutrients, such as vitamins and minerals, although required in smaller quantities, play crucial roles as enzymatic cofactors and regulators of numerous biochemical pathways; (iii) natural bioactive compounds (NBCs), substances found in plant-based foods (including polyphenols, carotenoids, phytosterols, and sulfur compounds) that exert protective effects thanks to their antioxidant and anti-inflammatory properties, contributing to the prevention of numerous chronic non-communicable diseases (CNCDs) [1,2,3,4].

Within the framework of the **15th anniversary issue of *Nutrients***, this multifaceted view of nutrition reflects the journal’s sustained focus over the past fifteen years on the integrated role of nutrients and bioactive compounds in human health and disease prevention. At the same time, important questions remain regarding the optimal balance, bioavailability, and long-term effects of these nutritional components across different populations and life stages—areas that continue to stimulate investigation.

In recent decades, scientific interest in NBCs has grown significantly, not only for their direct effects on host tissues but also for their ability to beneficially modulate gut microbiota [5]. This complex microbial ecosystem, consisting of trillions of microorganisms, plays an essential role in the digestion of indigestible nutrients, the synthesis of bioactive metabolites, and the regulation of the immune system. Specifically, NBCs and dietary fiber serve as substrates for microbial fermentation, promoting the growth of beneficial species and the production of short-chain fatty acids (SCFAs), such as acetate, propionate, and butyrate. These metabolites are essential for maintaining the integrity of the gut barrier, modulating the inflammatory response, and controlling energy metabolism [6,7,8,9]. Nevertheless, key questions remain regarding causality versus association, the identification of robust microbial biomarkers, and the development of targeted microbiota-based interventions.

The balance of the gut microbiota, known as eubiosis, is strongly influenced by dietary habits and lifestyle. A varied diet rich in plant-based foods promotes greater microbial diversity, considered an indicator of intestinal health [6]. Conversely, unhealthy dietary habits can lead to dysbiosis, characterized by a reduction in beneficial microorganisms and an increase in potentially pathogenic species. Numerous studies have highlighted a link between gut dysbiosis and the development of CNCDs, including obesity, type 2 diabetes, metabolic syndrome, arterial hypertension, chronic kidney disease, and cardiovascular disease. Furthermore, dysbiosis has been associated with increased intestinal permeability, resulting in the translocation of bacterial endotoxins and the activation of systemic inflammatory responses [10,11,12].

In this context, maintaining a healthy gut microbiota represents a crucial objective for the primary prevention of CNCDs. This can be achieved in a number of ways, such as through a balanced diet rich in fiber and NBCs, the intake of probiotics and prebiotics, and the adoption of a healthy lifestyle, including regular physical activity and a reduction in risk factors such as smoking and a sedentary lifestyle—key themes consistently emphasized in the journal’s contributions over its 15-year history. Future research directions include the personalization of dietary recommendations based on microbiome profiles and the long-term efficacy of combined lifestyle interventions.

In recent years, profound changes in the lifestyle and dietary patterns of Western populations have contributed to a significant increase in the incidence of CNCDs. The widespread adoption of the so-called Western diet, characterized by high calorie intake, abundant simple sugars, saturated fats, animal proteins, and ultra-processed foods, represents one of the main modifiable risk factors. This dietary pattern is often low in fiber, resulting in negative effects on both metabolism and the composition of gut microbiota [13,14,15]. While its detrimental effects are well documented, further investigation is needed to better understand the mechanisms linking specific components of ultra-processed foods to microbiota alterations and systemic inflammation. Specifically, the Western diet is associated with a reduction in microbial diversity and an increase in the prevalence of pro-inflammatory bacteria, resulting in a state of dysbiosis. This condition promotes increased intestinal permeability, which allows lipopolysaccharides and other pro-inflammatory molecules to enter the systemic circulation. This process contributes to the activation of the immune system and the development of chronic low-grade inflammation, recognized as one of the main pathogenic mechanisms underlying CNCDs [16,17,18]. These findings are consistent with a substantial body of evidence published in *Nutrients* over the years, underscoring the detrimental impact of modern dietary patterns.

In contrast, the Mediterranean diet is considered a healthy dietary pattern, widely validated by the scientific literature. It is characterized by a high consumption of fruits, vegetables, legumes, whole grains, nuts, and extra virgin olive oil (EVOO), a moderate intake of fish, and a low consumption of red meat and ultra-processed products. This dietary pattern ensures a high intake of fiber, monounsaturated fatty acids, and NBCs, which act synergistically to promote metabolic and intestinal health [19]. Within the scope of the *Nutrients* anniversary issue, the Mediterranean diet represents one of the most extensively studied and emblematic models of healthy eating, yet questions remain regarding its adaptability across different cultural contexts and its implementation at the population level.

Additionally, the high fiber and polyphenol content of the Mediterranean diet promotes the growth of beneficial bacteria, such as butyrate-producing bacteria, and stimulates the production of SCFAs. These metabolites have anti-inflammatory effects, improve intestinal barrier function, and contribute to the regulation of glucose and lipid metabolism. Furthermore, polyphenols exert direct antioxidant effects and modulate cellular signaling pathways involved in the inflammatory response [20,21,22,23]. Additional research is warranted to better define the relative contribution of individual dietary components versus overall dietary patterns. Epidemiological and clinical evidence indicates that a good adherence to the Mediterranean diet is associated with a significant reduction in the risk of developing CNCDs. These beneficial effects are mediated, at least in part, by modulating the gut microbiota and reducing systemic inflammation [24].

In conclusion, nutrition is a key factor in regulating the body’s homeostasis and preventing CNCDs. Therefore, adopting healthy dietary patterns represents an effective and sustainable strategy for the primary prevention of CNCDs and for improving quality of life. In the context of the **15th anniversary of *Nutrients***, these insights collectively highlight both the substantial progress achieved and the critical gaps that remain. Addressing these open questions—ranging from mechanistic understanding to personalized nutrition and public health implementation—will be essential for advancing the field. In this regard, *Nutrients* is uniquely positioned to continue serving as a leading platform for interdisciplinary research, fostering innovation and guiding future directions in nutrition science.
